# Robot-Assisted Extravesical Ureteral Reimplantation (RALUR-EV) in Children: Initial Single-Center Experience at a Public Tertiary-Care Hospital in Ecuador

**DOI:** 10.3390/jcm14228120

**Published:** 2025-11-17

**Authors:** Giancarlo Sánchez-Salazar, Juan Cruz-Álvarez, Pablo Guamán-Ludeña, Alice Gaibor-Pazmiño, Esteban Ortiz-Prado, Juan S. Izquierdo-Condoy

**Affiliations:** 1 Departamento de Cirugía Pediátrica, Hospital Carlos Andrade Marín, Quito 170103, Ecuador; 2 One Health Research Group, Universidad de Las Américas, Quito 170124, Ecuador

**Keywords:** vesicoureteral reflux, robot-assisted extravesical ureteral reimplantation, pediatric urology, minimally invasive surgery

## Abstract

**Introduction**: Vesicoureteral reflux (VUR) is a frequent pediatric urological anomaly associated with recurrent urinary tract infections and renal scarring. Evidence on robot-assisted extravesical ureteral reimplantation (RALUR-EV) continues to grow; however, reports from Latin America remain limited. **Objectives**: We report an initial single-center experience with transperitoneal RALUR-EV (Lich–Gregoir) in a public pediatric hospital in Ecuador, detailing operative metrics, perioperative outcomes, and short-term radiographic efficacy using standardized definitions. **Methods**: A retrospective, observational study was conducted at a public tertiary referral center in Quito (January 2021–May 2025). Consecutive children (0–17 years) with VUR or ureterovesical junction (UVJ) obstruction who underwent RALUR-EV with the Lich Gregoir technique were included. The primary outcome was radiographic resolution of VUR at 3–6 months on voiding cystourethrogram. Secondary outcomes were operative times (total, console, docking), length of stay, postoperative UTI (culture-confirmed), and complications (Clavien–Dindo). Analyses were descriptive; success was reported as both evaluable-only and intention-to-treat (ITT). The study received institutional ethics approval. **Results**: Nine children were included (median age 4.4 years; 5 girls). Eight had VUR (5 unilateral, 3 bilateral); one had isolated UVJ obstruction. Procedures were left-sided in 7 cases and right-sided in 2. Median total operative time was 135 min (IQR 129–153); median console and docking times were 120 and 15 min, respectively. No intraoperative complications or conversions occurred. Median length of stay was 4 days (IQR 3–4). Two culture-confirmed postoperative UTIs occurred (2/9; Clavien II); no complications ≥ III were observed. Postoperative imaging was available in 6/9 cases (66.7%): radiographic resolution was 6/6 (100%) overall and 5/5 (100%) among VUR-only. ITT success was 6/9 (66.7%) overall and 5/8 (62.5%) for VUR-only. **Conclusions**: Transperitoneal RALUR-EV is feasible and safe in a public tertiary setting, with early effectiveness comparable to international series. Standardized pathways, structured follow-up, and multicenter collaboration are warranted to confirm durability and support broader regional adoption.

## 1. Introduction

Vesicoureteral reflux (VUR) is among the most common congenital urological anomalies in children and is associated with recurrent urinary tract infections (UTIs), renal scarring, and long-term kidney damage [1]. Its prevalence is estimated at 1–2% in the general pediatric population and rises to 30–50% among children presenting with febrile UTIs [2]. The principal goal of VUR management is preservation of renal function through prevention of recurrent infection and ongoing reflux [3].

Management options range from continuous antibiotic prophylaxis to open ureteral reimplantation. Open techniques—including the Cohen and Lich–Gregoir methods—achieve success rates exceeding 95% but are typically accompanied by greater postoperative pain, longer hospital stays, and more visible scars [4,5]. Advances in minimally invasive surgery have expanded therapeutic choices to laparoscopic and robot-assisted approaches, which can offer improved cosmesis, reduced postoperative pain, and shorter convalescence [6]. Robot-assisted laparoscopic ureteral reimplantation (RALUR), particularly using the extravesical Lich–Gregoir technique (RALUR-EV), avoids cystotomy and may reduce hematuria and bladder spasms [7,8]. Multiple series have reported high radiographic success with low complication rates in pediatric cohorts [6,7,8,9]. However, most evidence originates from North America, Europe, and parts of Asia, with limited data from Latin America.

In Latin America, the adoption of robotic pediatric urology remains limited due to structural, economic, and training barriers. Most robotic platforms are concentrated in adult surgical centers and private institutions, resulting in a lack of equitable access for children treated in public hospitals [10,11]. For instance, a regional survey reported that only ten public institutions since 2009 had acquired robotic systems and half of them later suspended their programs because of high operational costs. Moreover, standardized outcome reporting and long-term follow-up data are scarce across the region [12]. Expanding regional evidence is therefore essential not only for clinical benchmarking but also to inform policymakers on cost-effectiveness and program sustainability in resource-limited healthcare systems [13,14]. The present work aims to address this gap by documenting early outcomes, safety metrics, and implementation strategies within a public tertiary hospital framework. This regional perspective provides a foundation for future multicenter collaborations and contributes to the global understanding of how pediatric robotic programs can be safely and efficiently implemented in developing countries.

## 2. Materials and Methods

### 2.1. Study Design and Setting

We conducted a retrospective, observational, and descriptive study at the Carlos Andrade Marín Specialty Hospital (HECAM), a public tertiary-care referral and specialty center of the Ecuadorian Social Security Institute (IESS) located in Quito, Ecuador [15]. The study period spanned January 2021 to May 2025, including all consecutive patients aged 0–17 years undergoing robot-assisted extravesical ureteral reimplantation using the Lich–Gregoir technique.

### 2.2. Participants

We included all consecutive patients aged 0–17 years with a confirmed diagnosis of vesicoureteral reflux (VUR) or ureterovesical junction (UVJ) obstruction who were treated surgically with a robotic approach during the study period. No predefined exclusions were applied.

### 2.3. Surgical Technique

Surgical setup and patient positioning followed standard pediatric robotic protocols. Patients 1 to 6 were operated using the Da Vinci Si robotic platform and patients 7 to 9 were operated using the Da Vinci Xi robotic platform, with patients placed supine and Trendelenburg position to optimize exposure. A transperitoneal three-port configuration was used in all cases (8 mm camera port at the umbilicus and two 8 mm working ports placed along the midclavicular line). When needed, an accessory 5 mm assistant port was introduced for surgical laparoscopic aspiration. Pneumoperitoneum was maintained at 10–12 mmHg with low-flow CO_2_ insufflation. After mobilizing the ureter extravesically, in the case of UVJ obstruction, a ureteral stent (JJ Stent) was placed into the ureter before suturing it with 5-0 polyglactin discontinuous suture, and in all cases detrusorotomy and submucosal tunneling were performed under direct vision according to the Lich–Gregoir technique. The detrusor was closed with a single-layer 3-0 polyglactin discontinuous suture, ensuring watertight coverage [16,17]. Perioperative antibiotic prophylaxis consisted of a single intravenous dose of cefazolin (30 mg/kg) administered 30 min before incision. Postoperative analgesia included acetaminophen and ketorolac (depending on age), without rescue morphine required. Foley catheters were maintained 24–48 h, and discharge criteria included afebrile status, adequate oral intake, and spontaneous voiding. Follow-up was standardized at 2 weeks for wound review, 1 month to remove the ureteral stent by cystoscopy (in the only case used ureteral stent) and at 3–6 months for radiographic assessment (VCUG). Operations were performed in all cases by the same pediatric surgeon formally trained in robotic surgery.

### 2.4. Variables and Definitions

The following clinical and perioperative variables were collected: age at surgery, sex, clinical diagnosis, VUR laterality and grade [18], total operative time, docking time, console time, length of hospital stay, the presence of postoperative urinary tract infection (UTI) [19], intraoperative or postoperative complications, and surgical success. Surgical success was defined as absence of symptomatic VUR or its absence on postoperative imaging (voiding cystourethrogram, VCUG), when available. UTIs were considered when clinically compatible and culture-confirmed; complications were summarized descriptively and, when applicable, categorized using the Clavien–Dindo classification [20,21].

### 2.5. Data Sources and Management

Data were extracted from electronic medical records, operative reports, anesthetic logs, and nursing progress notes, and consolidated in a secure Microsoft Excel database created for the study. All data were de-identified prior to analysis; no direct personal identifiers (e.g., names, national ID numbers, exact addresses, contact details) were collected or retained. Data quality checks included range and consistency verification prior to analysis, and access to the working dataset was restricted to the study team.

### 2.6. Statistical Analysis

Given the descriptive nature and small sample, analyses were limited to descriptive measures, using counts and percentages for categorical variables, and medians with interquartile ranges (IQR) or means with standard deviations (SD) for continuous variables, as appropriate. No hypothesis testing was planned. For outcome reporting, we present both an intention-to-treat success proportion (using the entire cohort as the denominator) and an evaluable-only success proportion (restricted to patients with postoperative imaging).

### 2.7. Ethical Considerations

The study protocol was approved by the institutional ethics committee and conducted in accordance with the ethical standards of the institutional research committee and the Declaration of Helsinki and its later amendments. HECAM issued an official institutional letter of support authorizing access to clinical data for research purposes. No identifiable personal data were collected or handled at any stage of the study, and patient confidentiality was preserved throughout data collection, management, and analysis.

## 3. Results

### 3.1. Cohort Characteristics

Between January 2021 and May 2025, nine children underwent robot-assisted extravesical ureteral reimplantation (Lich–Gregoir). The median age at surgery was 4.4 years (IQR 2.8–9.3; mean 6.0; range 1.3–13.5), and 5/9 (55.6%) were female. Overall, 8/9 (88.9%) had vesicoureteral reflux (VUR) and 1/9 (11.1%) had ureterovesical junction (UVJ) obstruction without VUR. Among patients with VUR (*n* = 8), 5/8 (62.5%) had unilateral and 3/8 (37.5%) had bilateral reflux. In bilateral VUR, only the ureter with clinically significant reflux (≥grade III) was reimplanted, and the contralateral low-grade reflux (grade I–II) was managed conservatively (Table 1).

### 3.2. Operative Metrics

**Figure 1:**
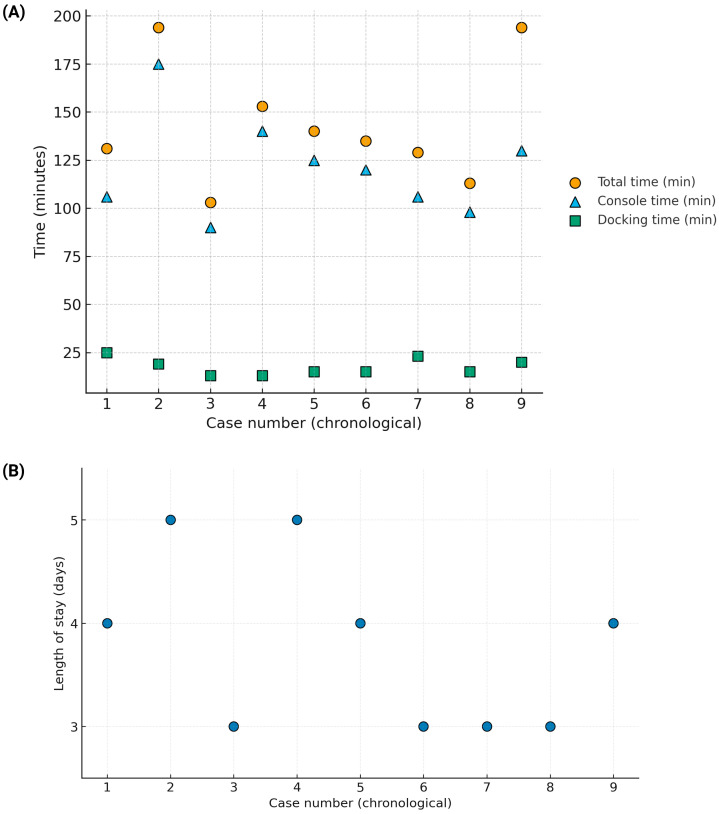
Operative times and hospital stay by case (chronological order). (**A**) Operative times by case. Scatter plot showing total, console, and docking times for each case in chronological order. Points are displayed without connecting lines to avoid implying a learning-curve trend. (**B**) Hospital stay by case. Scatter plot showing the length of stay (days) per case in chronological order.

Reimplantation was left-sided in 7/9 (77.8%) of cases and right-sided in 2/9 (22.2%). The median total operative time was 135 min (IQR 129–153; mean 143.6; range 103–194), with a median console time of 120 min (IQR 106–130; mean 121.1) and a median docking time of 15 min (IQR 15–20; range 13–25). There were no intraoperative complications and no conversions to open surgery. Operative times and length of stay by case are visualized in Figure 1A,B and detailed numerically in Table 1.

### 3.3. Perioperative Outcomes

The median hospital stay was 4 days (IQR 3–4; mean 3.8; range 3–5). Two patients developed culture-confirmed postoperative UTIs (2/9; 22.2%), both Clavien–Dindo II, managed with oral antibiotics; no complications ≥ Clavien–Dindo III were recorded (Table 1).

### 3.4. Follow-Up and Primary Outcome

At 3–6 months, 6/9 (66.7%) patients had postoperative imaging available. Of these, radiographic resolution was confirmed in 6/6 (100%); restricting to VUR-only cases, 5/5 (100%) had radiographic resolution of reflux. Three additional patients (3/9; 33.3%) were pending imaging at the time of analysis and remained asymptomatic (Table 1). For transparency, we also report evaluable-only success 6/6 (100%) overall; intention-to-treat success 6/9 (66.7%) overall; VUR-only: 5/5 (100%) evaluable vs. 5/8 (62.5%) ITT.

## 4. Discussion

This single-center experience with RALUR-EV in a public pediatric hospital in Ecuador indicates that the procedure is feasible and safe within a resource-constrained health system. Among nine children, there were no intraoperative complications and no conversions to open surgery. Median length of stay was 4 days (IQR 3–4), and two culture-confirmed postoperative UTIs (2/9; Clavien–Dindo II) were managed conservatively with oral antibiotics, with no complications ≥ III recorded. For effectiveness, radiographic resolution among evaluable patients was 6/6 (100%) overall and 5/5 (100%) for VUR-only. Using an intention-to-treat denominator to account for pending imaging, overall success was 6/9 (66.7%), and 5/8 (62.5%) for VUR-only. Reporting both evaluable and intention-to-treat proportions provides transparency about early outcomes and follow-up completeness.

Our radiographic success rate (100% among evaluable patients; 66.7% by intention-to-treat) is consistent with previously published RALUR-EV series reporting success rates between 85% and 100%, depending on reflux grade, surgical experience, and institutional volume [6,22,23]. The absence of intraoperative complications or conversions further supports the safety profile of RALUR-EV, even within a low-volume, resource-constrained public setting. This finding mirrors that of Sforza et al. [24], who reported complication rates below 5% across multiple European centers, suggesting that standardized technique and perioperative pathways can mitigate the variability associated with limited caseloads. Similarly, the low incidence of postoperative UTI in our cohort aligns with contemporary data, reinforcing that RALUR-EV achieves safety outcomes comparable to open and laparoscopic reimplantation when performed by trained pediatric robotic surgeons [23].

Operative times in our cohort were slightly longer than those reported by high-volume centers (typically 90–120 min) [22,25], which likely reflects the early learning phase of our institutional robotic program and the limited number of cases performed [26]. Several studies have demonstrated that after approximately 10–15 RALUR-EV procedures, operative efficiency and outcomes reach a plateau comparable to open surgery benchmarks [22,27]. Progressive reductions in docking and console times are therefore expected as experience accumulates, and workflow optimization continues. Although our chronological case display did not demonstrate a clear downward trend, continuous procedural standardization and increasing surgical volume are anticipated to improve efficiency. Notably, our short median hospital stay (4 days) suggests that early discharge—and potentially same-day discharge—is feasible under structured perioperative pathways, as reported in other contemporary series [7,22].

However, this preliminary experience has inherent limitations. The study’s small sample size and single-center design limit statistical power and external validity. Incomplete postoperative imaging in one-third of patients may underestimate true success, although ongoing follow-up is underway. In addition, no patient-reported outcomes (such as pain, satisfaction, or recovery metrics) or cost-effectiveness analyses were included—elements increasingly recognized as essential for comprehensive pediatric surgical assessment [27,28]. These factors should be addressed in future multicenter studies with standardized follow-up and integration of patient-centered and economic endpoints to strengthen generalizability and inform sustainable program implementation.

This approach also supports the feasibility of pursuing early-discharge pathways in selected patients, as previously reported [7].

From a regional perspective, this experience complements the early Latin-American report by Garibay et al. from Mexico [8]. and constitutes, to our knowledge, the first published pediatric robotic urology series from Ecuador, contributing context-specific evidence to a sparse regional literature. Persistent structural barriers—capital and maintenance costs of robotic platforms, limited public coverage, and low case volumes that constrain skill acquisition—remain significant in Latin America [9]. Our service mitigated these constraints by integrating pediatric cases into an established adult robotic program, enabling shared infrastructure, instruments, and trained staff. This shared-platform model may be replicable for public hospitals across the region facing similar fiscal and workforce limitations.

Recent bibliometric analyses indicate that, although robotic surgery publications in Latin America have grown steadily since 2009, over 80% of robotic platforms remain concentrated in private or mixed institutions, and pediatric public-sector experiences are exceptionally scarce. This underscores the contextual relevance of our findings and the importance of documenting early implementation within public healthcare systems in the region [11].

From a health-system perspective, the principal challenge for replicating RALUR-EV in public hospitals lies in the substantial upfront investment and ongoing maintenance costs of robotic platforms. Emerging evidence indicates that cost-effectiveness improves when robotic systems are shared across surgical specialties and when enhanced perioperative outcomes reduce hospitalization and complication-related expenses [29,30,31,32]. Within this context, our shared pediatric–adult robotic infrastructure represents a pragmatic framework for Latin American public hospitals, balancing technological innovation with financial sustainability. Future regional studies should incorporate formal cost–utility and cost-minimization analyses to provide policymakers with robust evidence on the economic feasibility and long-term value of expanding pediatric robotic programs.

In children with bilateral VUR, our practice of reimplanting only the clinically significant side (≥grade III) and managing contralateral low-grade reflux (I–II) conservatively is consistent with contemporary selection criteria and with efforts to limit morbidity associated with bilateral extravesical dissection. Given the theoretical risk of transient urinary retention after bilateral extravesical reimplantation, a selective unilateral approach appears reasonable during program ramp-up, provided that the contralateral side is monitored closely during follow-up [9].

To consolidate and responsibly scale pediatric robotics in the region, we recommend: (i) standardized perioperative pathways (selection criteria, analgesia, antibiotic prophylaxis, discharge criteria); (ii) structured follow-up with uniform imaging at 3–6 months to minimize loss to follow-up and enable consistent endpoint reporting; (iii) multicenter collaborations and a regional registry to study learning curves, safety, and benchmarking; (iv) integration of patient-centered outcomes and economic evaluations to inform decision-makers; and (v) expansion of training programs (simulation, visiting fellowships), ideally supported by shared platforms with adult services to optimize utilization and sustainability [9]. Taken together, these steps should accelerate program maturation, improve efficiency, and facilitate outpatient pathways (including same-day discharge) in carefully selected cases [7].

Economic evaluations in adult urologic robotic procedures demonstrate that robotics can decrease postoperative morbidity and hospital stay, yet often at higher direct costs; cost-effectiveness improves mainly in high-volume centers or shared-platform models [29,30,33]. Although pediatric-specific economic data remain limited, our institutional framework—integrating pediatric cases within an established robotic program—can distribute fixed costs, optimize personnel utilization, and enhance sustainability in a public healthcare environment. Future regional studies should incorporate formal cost–utility and budget–impact analysis to guide evidence-based allocation of resources.

Future research should expand regional evidence through multicenter registries using standardized outcome definitions and extended follow-up to assess reflux recurrence and renal preservation. Integration of patient-reported outcomes—including pain, satisfaction, and cosmetic perception, together with economic and quality-of-life assessments will be essential to ensure equitable implementation in public healthcare systems with constrained budgets. Furthermore, establishing regional training networks that combine simulation-based curricula and fellowships is critical to building long-term pediatric robotic expertise in Latin America.

Ultimately, the successful establishment of RALUR-EV within a public tertiary institution in Ecuador demonstrates that advanced minimally invasive pediatric surgery can transcend traditional resource barriers when guided by structured protocols, regional collaboration, and institutional commitment. This experience not only expands the geographic and socioeconomic boundaries of pediatric robotic urology but also serves as a blueprint for sustainable innovation in developing healthcare systems. By coupling technological progress with equitable access and rigorous outcome reporting, Latin America can help redefine the global narrative of pediatric surgical advancement [33].

### Limitations

This study was retrospective, single-center, and included a small sample, limiting precision and external validity. It reflects the early phase of our institutional robotic program, where results may still be influenced by the learning curve. Incomplete postoperative imaging required reporting both intention-to-treat and evaluable-only outcomes, and the true success rate may be higher once pending studies are completed. As a public tertiary experience from Ecuador, generalizability to other regional settings is limited. Patient-reported outcomes and economic evaluations were not performed, restricting a comprehensive assessment of recovery, satisfaction, and cost implications. Although complications were classified using the Clavien–Dindo system, the limited sample precludes meaningful comparison of rare adverse events. Future multicenter studies with standardized follow-up, inclusion of patient-centered metrics, and long-term evaluation of program sustainability are warranted.

## 5. Conclusions

RALUR-EV, Lich–Gregoir was feasible and safe in our public tertiary setting, with no intraoperative complications or conversions, a median hospital stay of 4 days, and two culture-confirmed UTIs (Clavien II). Effectiveness was high among evaluable patients (6/6 overall; 5/5 VUR-only radiographic resolution), while intention-to-treat reporting (6/9 overall; 5/8 VUR-only) transparently reflects pending imaging. These early outcomes are broadly consistent with international series and constitute, to our knowledge, the first pediatric robotic urology report from Ecuador, adding context-specific evidence from Latin America.

Scaling this approach responsibly will require standardized perioperative pathways, structured 3–6-month imaging follow-up, and targeted training—ideally leveraging shared platforms with adult services to optimize utilization and costs. Given the small, single-center design and incomplete radiographic follow-up, multicenter collaborations and regional registries are needed to confirm durability, refine indications, and evaluate cost-effectiveness. With institutional support and programmatic learning, outpatient pathways (including same-day discharge in selected cases) may be achievable, helping extend the benefits of advanced minimally invasive surgery to more children across the region.

## Figures and Tables

**Table 1:** Summary of patient characteristics and perioperative data.

Surgery Date	Diagnosis	Procedure	Total (min)	Docking (min)	Console (min)	Age	Sex	VUR Grade	Success	Postop UTI	Complications	Stay (Days)
7 December 2021	Left VUR	Left reimplant	131	25	106	2 years, 9 months	M	III	Yes	No	No	4
18 July 2022	Right UVJ obstruction	Right reimplant	194	19	175	13 years, 6 months	M	—	Yes	Yes	Clavien–Dindo II	5
7 November 2022	Left VUR	Left reimplant	103	13	90	4 years, 5 months	M	III	Yes	No	No	3
28 November 2022	Left VUR	Left reimplant	153	13	140	1 year, 4 months	F	III	Yes	Yes	Clavien–Dindo II	5
6 March 2023	Left VUR	Left reimplant	140	15	125	9 years, 3 months	F	III	Yes	No	No	4
16 December 2024	Bilateral VUR	Left reimplant	135	15	120	11 years, 2 months	F	R: I/L: III	Yes	No	No	3
13 January 2025	Bilateral VUR	Left reimplant	129	23	106	2 years, 2 months	F	R: II/L: III	—	No	No	3
25 April 2025	Left VUR	Left reimplant	113	15	98	6 years, 7 months	F	III	—	No	No	3
19 May 2025	Bilateral VUR	Right reimplant	150	20	130	3 years, 0 months	M	R: IV/L: I	—	No	No	4

Abbreviations: VUR, vesicoureteral reflux; UVJ, ureterovesical junction; R, right; L, left. “—” indicates not available or follow-up pending. Note: For UVJ obstruction, Success denotes clinical/radiographic resolution of obstruction; for VUR, Success denotes radiographic resolution of reflux (VCUG).

## Data Availability

All data generated or analyzed during this study are included in this published article.

## References

[1] Läckgren G., Cooper C.S., Neveus T., Kirsch A.J. (2021). Management of Vesicoureteral Reflux: What Have We Learned Over the Last 20 Years?. Front. Pediatr..

[2] Finnell S.M.E., Carroll A.E., Downs S.M., the Subcommittee on Urinary Tract Infection (2011). Diagnosis and Management of an Initial UTI in Febrile Infants and Young Children. Pediatrics.

[3] Campbell Walsh Wein Urology-9780323546423. MEA Elsevier Health. https://www.eu.elsevierhealth.com/campbell-walsh-wein-urology-9780323546423.html.

[4] Gnech M., ’t Hoen L., Zachou A., Bogaert G., Castagnetti M., O’Kelly F., Quaedackers J., Rawashdeh Y.F., Silay M.S., Kennedy U. (2024). Update and Summary of the European Association of Urology/European Society of Paediatric Urology Paediatric Guidelines on Vesicoureteral Reflux in Children. Eur. Urol..

[5] Peters C.A., Skoog S.J., Arant B.S., Copp H.L., Elder J.S., Hudson R.G., Khoury A.E., Lorenzo A.J., Pohl H.G., Shapiro E. (2010). Summary of the AUA Guideline on Management of Primary Vesicoureteral Reflux in Children. J. Urol..

[6] Esposito C., Masieri L., Steyaert H., Escolino M., Cerchione R., La Manna A., Cini C., Lendvay T.S. (2018). Robot-assisted extravesical ureteral reimplantation (revur) for unilateral vesico-ureteral reflux in children: Results of a multicentric international survey. World J. Urol..

[7] Chertin L., Kocherov S., Bakaleyshchik P., Baranov Y., Dubrov V., Kagantsov I., Karpachev S., Kuzovleva G., Pirogov A., Rudin Y. (2024). Laparoscopic and Robot-assisted Laparoscopic Reimplantation for Lower Ureter Pathology. A Multi-institutional Comparative Study in 1343 Patients. Urology.

[8] Babajide R., Andolfi C., Kanabolo D., Wackerbarth J., Gundeti M.S. (2023). Postoperative hydronephrosis following ureteral reimplantation: Clinical significance and importance of surgical technique and experience. J. Pediatr. Surg..

[9] Boysen W.R., Ellison J.S., Kim C., Koh C.J., Noh P., Whittam B., Palmer B., Shukla A., Kirsch A., Gundeti M.S. (2017). Multi-Institutional Review of Outcomes and Complications of Robot-Assisted Laparoscopic Extravesical Ureteral Reimplantation for Treatment of Primary Vesicoureteral Reflux in Children. J. Urol..

[10] Aucatoma F.C., Pazmiño M.C.B., Ludeña P.G. (2022). Características clínicas y resultados quirúrgicos de pacientes pediátricos intervenidos por cirugía robótica. Rev. Médica-Cient. CAMbios HECAM.

[11] Rivero-Moreno Y., Cordova-Guilarte J., Echevarria S., Dorado-Avila G., Pianetti L., Acevedo-Rodríguez J., Chavez-Campos C., Paz-Castillo-Lopez M., Estrella-Gaibor C., Salcedo Y. (2023). Innovation in Motion: Robotic Surgery’s status in Latin America. Ambul. Surg..

[12] Secin F.P., Coelho R., Monzó Gardiner J.I., Salcedo J.G.C., Puente R., Martínez L., Finkelstein D., Valero R., León A., Angeloni D. (2018). Robotic surgery in public hospitals of Latin-America: A castle of sand?. World J. Urol..

[13] Garibay González F., Navarrete Arellano M., Castillo Niño J.C., García González F.M., Sánchez Alejo J.A. (2018). Robotic surgery in urology. First prospective pediatric case series in Latin America. Rev. Sanid. Mil..

[14] SciELO Brasil-Robotics in Pediatric Urology Robotics in Pediatric Urology. https://www.scielo.br/j/ibju/a/ndRDtJL55DN4pfStbtQkYyw/?format=html&lang=en.

[15] Hospital de Especialidades Carlos Andrade Marín (2024). Quienes Somos—Hospital Carlos Andrade Marín. Hospital de Especiali-dades Carlos Andrade Marín. https://hcam.iess.gob.ec/quienes-somos/.

[16] Bustangi N., Kallas Chemaly A., Scalabre A., Khelif K., Luyckx S., Steyaert H., Varlet F., Lopez M. (2018). Extravesical Ureteral Reimplantation Following Lich-Gregoir Technique for the Correction of Vesico-Ureteral Reflux Retrospective Comparative Study Open vs. Laparoscopy. Front. Pediatr..

[17] Mei H., Tang S. (2023). Robotic-assisted surgery in the pediatric surgeons’ world: Current situation and future prospectives. Front. Pediatr..

[18] Lebowitz R.L., Olbing H., Parkkulainen K.V., Smellie J.M., Tamminen-Möbius T.E. (1985). International system of radiographic grading of vesicoureteric reflux. Pediatr. Radiol..

[19] ’t Hoen L.A., Bogaert G., Radmayr C., Dogan H.S., Nijman R.J.M., Quaedackers J., Rawashdeh Y.F., Silay M.S., Tekgul S., Bhatt N.R. (2021). Update of the EAU/ESPU guidelines on urinary tract infections in children. J. Pediatr. Urol..

[20] Dindo D., Demartines N., Clavien P.-A. (2004). Classification of Surgical Complications. Ann. Surg..

[21] Clavien P.A., Barkun J., de Oliveira M.L., Vauthey J.N., Dindo D., Schulick R.D., de Santibañes E., Pekolj J., Slankamenac K., Bassi C. (2009). The Clavien-Dindo classification of surgical complications: Five-year experience. Ann. Surg..

[22] Essamoud S., Ghidini F., Andolfi C., Gundeti M.S. (2024). Robot-assisted laparoscopic extravesical ureteral reimplantation (RALUR-EV): A narrative review. Transl. Pediatr..

[23] Hou S.W., Xing M.H., Gundeti M.S. (2023). Pediatric robotic urologic procedures: Indications and outcomes. Indian J. Urol..

[24] Sforza S., Marco B.B., Haid B., Baydilli N., Donmez M.I., Spinoit A.-F., Paraboschi I., Masieri L., Steinkellner L., Comez Y.I. (2024). A multi-institutional European comparative study of open versus robotic-assisted laparoscopic ureteral reimplantation in children with high grade (IV–V) vesicoureteral reflux. J. Pediatr. Urol..

[25] Neheman A., Strine A.C., Concodora C.W., Schulte M.E., Noh P.H. (2019). Outpatient Robotic Unilateral Extravesical Ureteral Reimplantation in the Pediatric Population: Short-Term Assessment of Safety. J. Urol..

[26] Kim J.K., Batra N., Shavnore R., Szymanski K.M., Misseri R., Kaefer M., Cain M.P., Roth J., Dangle P., Meldrum K. (2025). Attaining competency and proficiency in pediatric robot-assisted laparoscopic ureteric reimplantation: A learning curve configuration using cumulative sum analysis. World J. Urol..

[27] Chen C.J., Peters C.A. (2019). Robotic Assisted Surgery in Pediatric Urology: Current Status and Future Directions. Front. Pediatr..

[28] Besner A.-S., Ferreira J.L., Ow N., Gaffar R., Guadagno E., Emil S., Poenaru D. (2022). Patient-reported outcome measures in pediatric surgery—A systematic review. J. Pediatr. Surg..

[29] Dixon S., Hill H., Flight L., Khetrapal P., Ambler G., Williams N.R., Brew-Graves C., Kelly J.D., Catto J.W.F., iROC Study Team (2023). Cost-Effectiveness of Robot-Assisted Radical Cystectomy vs Open Radical Cystectomy for Patients with Bladder Cancer. JAMA Netw. Open.

[30] Hong Y.E., Shim H., Shin M. (2025). Costs and cost-effectiveness of robotic-assisted surgery in South Korea: A systematic review and meta-analysis. Front. Public Health.

[31] Tang Y., Dou B. (2025). Cost-effectiveness analysis of robotic surgery in healthcare for older individuals: A systematic review based on randomized controlled trials. Front. Public Health.

[32] Lai T.-J., Heggie R., Kamaruzaman H.-F., Bouttell J., Boyd K. (2025). Economic Evaluations of Robotic-Assisted Surgery: Methods, Challenges and Opportunities. Appl. Health Econ. Health Policy.

[33] Lai T.-J., Roxburgh C., Boyd K.A., Bouttell J. (2024). Clinical effectiveness of robotic versus laparoscopic and open surgery: An overview of systematic reviews. BMJ Open.

